# Dietary supplementation of *Ascophylum nodosum* improved kidney function of mink challenged with Aleutian mink disease virus

**DOI:** 10.1186/s12917-020-02685-w

**Published:** 2020-11-30

**Authors:** A. Hossain Farid, Nancy J. Smith

**Affiliations:** 1 Department of Animal Science and Aquaculture, Faculty of Agriculture, Dalhousie University, Truro, Nova Scotia B2N 5E3 Canada; 2 Perennia Food and Agriculture, Bible Hill, Nova Scotia B4N 1J5 Canada; 3 Present Address: Farm Credit Canada, 332 Willow St, Truro, Nova Scotia B2N 5A5 Canada

**Keywords:** Aleutian mink disease virus; American mink, *Ascophylum nodosum*, Kidney function, Serum profile

## Abstract

**Background:**

Feed additives which can ease the negative effects of infection by the Aleutian mink disease virus (AMDV) are of interest to mink farmers. The effects of kelp meal (*Ascophylum nodosum*) supplementation on immune response, virus replication and blood parameters of mink inoculated with AMDV were assessed. AMDV-free black mink (*n* = 75) were intranasally inoculated with a local strain of AMDV and fed a commercial pellet supplemented with kelp meal at the rates of 1.5% or 0.75% of the feed or were kept as controls (no kelp) for 451 days. Blood was collected on days 0 (pre-inoculation), 31, 56, 99, 155, 366 and 451 post-inoculation (dpi).

**Results:**

No significant difference was observed among the treatments for the proportion of animals positive for antibodies against the virus measured by the counter-immunoelectrophoresis (CIEP), viremia measured by PCR, antibody titer measured by quantitative ELISA, total serum protein measured by a refractometer or elevated levels of gamma globulin measured by iodine agglutination test at the sampling occasions. At the termination of the experiment on 451 dpi, there were no differences among treatments for antibody titer measured by CIEP, total serum protein, albumin, globulins, albumin:globulin ratio, alkaline phosphatase, gamma-glutamyl transferase, and proportions of PCR positive spleen, lymph node or bone marrow samples, but blood urea nitrogen and creatine levels were significantly lower in the 1.5% kelp supplemented group than in the controls.

**Conclusion:**

Kelp supplementation improved kidney function of mink infected with AMDV with no effect on liver function, immune response to infection by AMDV or virus replication.

## Background

AMDV causes a serious health problem for farmed mink globally. Infected mink show persistent antiviral antibody production, hypergammaglobulinemia, general plasmacytosis and progressive renal disease, leading to death in some mink [1]. The virus is very resilient and remains infectious in composted materials [2]. AD has no cure or a vaccine [3], and almost 40 years of elimination of seropositive animals from herds has not been effective in controlling the infection on many farms in Canada [4] and other countries [5]. Consequently, selection of mink for tolerance to AMDV has gained popularity in Canada and some other countries in recent years. Keeping infected mink to evaluate their degree of tolerance increases mortality during the initial phases of this undertaking. Any treatment that can improve the health and survival of mink on infected farms is of interest to farmers.

Recent in vitro studies revealed that seaweed extracts have anti-herpes simplex virus (HSV-1, NSV-2) [6, 7], anti-human immunodeficiency virus (HIV-1) [7, 8], anti-chikungunya virus [9], anti-enterovirus (ECHO-1) [7] properties and inactivated measles virus [10] to name a few. Extensive reviews suggest that seaweed extracts can block virus-host attachment, virus entry to the cell or inhibit the replication of several types of enveloped viruses in vitro [11–16]. The substances which have antiviral properties in vitro must eventually be tested in vivo prior to approval for use in animals or humans. Difficulties in keeping infected animals and in performing clinical trials in humans have resulted in a limited number of in vivo studies on antiviral effect*s* of seaweed extracts. Dietary supplementation of mice with crude sulfated galactans derived from red seaweeds [17], and from brown seaweed extracts [7] showed antiviral activity against vaginal infection with herpes simplex virus types 1 and 2, likely as a result of an inhibitory effect on virus adsorption [17]. A green seaweed extract reduced adsorption and blocked some early steps of the enterovirus 71 (EV71) life cycle in vitro, and its intramuscular injection markedly reduced virus titers in infected mice [18]. It may be concluded that substances which show positive antiviral effects in vitro also show positive effects in vivo. Although published reports mostly cover enveloped virus for which effective vaccines are not available, and there is no published information on the non-enveloped DNA viruses, such as AMDV, logically seaweeds could influence AMDV pathogenesis as well. This assumption is based on the evidence that seaweed extracts have immunomodulatory effects [19, 20], which play an important role in AMDV-pathogenesis [1].

The objectives of this study were to assess the effects of dietary supplementation of kelp *(Ascophyllum nodosum)* on immune response of, and virus replication in mink challenged with AMDV. Kelp was used in this study because it grows in great quantities along the shores of the North Atlantic, including Nova Scotia, Canada, and has a long history of usage as livestock feed [21]. This is a novel study because there is no in vivo study on the antiviral effects of seaweed meals rather than their specific extracts, because various seaweed constituents could have differential effects on virus entry to the cell, virus establishment and propagation, as well as on the fate of the virus within the animal’s body in response to the host immune response and viral clearance.

## Results

### Viral DNA in the blood and tissues

**Fig. 1:**
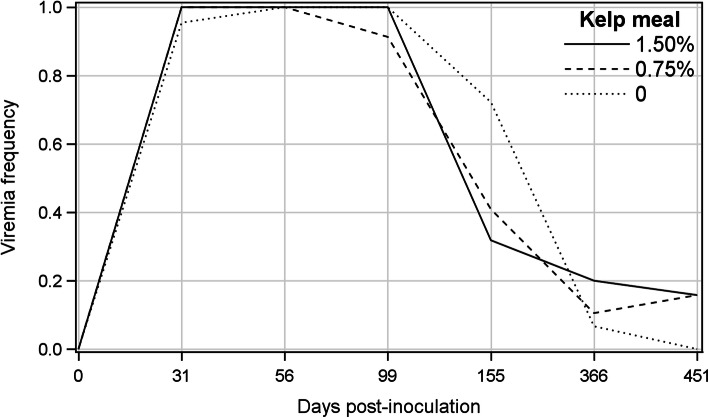
Frequency of viremic mink of the kelp-supplemented and control groups on different post-inoculation days

Viral DNA was detected by PCR in the blood of all animals by 31 dpi, except in one mink which became PCR positive by 56 dpi. The incidence of viremia steadily declined after 56 dpi (Fig. 1), and the averages of the number of viremic mink over all treatments were 0.97, 0.47, 0.13 and 0.11 at 99, 155, 366 and 451 dpi, respectively. Five mink remained viremic throughout the experiment and one non-viremic mink from T0.75 treatment at 366 dpi became viremic at 451 dpi. The GENMOD analysis showed no significant treatment or treatment by sampling day interaction, and the odds ratios for the incidence of viremia were comparable among treatments (Table 1). The rate of decline in the proportion of PCR positive animals over time was significant (β = − 1.811) showing that the odds of decrease in PCR positive cases for each unit increment in sampling occasion was large (odds ratio = 0.163). Differences among treatments for the proportion of PCR-positive blood, spleen, lymph node and bone marrow samples at the termination of the experiment were not significant (Table 2).

**Table 1:** The GEE parameter estimates, 95% confidence limits and odds ratios for the prevalence of viremia measured by PCR, seropositive measured by CIEP and IAT positive results in the blood from day 31 to 451 post-inoculation

Measurement	Viremia	Seropositive	IAT (0,1)	IAT score (0–4)
Analysis^a^				
Treatment	1.82 (0.40)	1.09 (0.58)	1.49 (0.48)	1.06 (0.58)
Sampling days	52.71 (< 0.01)	4.79 (0.03)	0.26 (0.61)	10.16(< 0.01)
Treatment X days	1.29 (0.52)	3.01 (0.21)	2.01 (0.37)	0.32 (0.85)
Estimates^b^				
1.5% kelp	−0.233 (−1.24, 0.77)	−0.110 (− 1.78,1.56)	−0.220 (− 1.17, 0.73)	−0.031 (− 0.76,0.70)
0.75% kelp	− 0.376 (− 1.247, 0.56)	−0.424 (− 2.08,1.23)	−0.624 (− 1.54, 0.29)	−0.470 (− 1.23, 0.29)
0% kelp	Reference	Reference	Reference	Reference
Sampling days	−1.811 (−2.29,-1.33)	0.120 (−0.01, 0.25)	0.028 (−0.21, 0.26)	− 0.185 (− 0.30, − 0.07)
Odds ratio^b^				
0.75–1.5%	0.866 (0.28–2.65)	0.731 (0.16–3.25)	0.668 (0.27–1.64)	0.644 (0.30–1.39)
0.75–0%	0.686 (0.27–1.75)	0.655 (0.13–3.42)	0.536 (0.21–1.34)	0.625 (0.29–1.33)
1.5–0%	0.792 (0.29–2.16)	0.896 (0.17–4.74)	0.803 (0.31–2.07)	0.969 (0.47–2.01)
Sampling days^b^	0.163 (0.10–0.26)	1.127 (0.98–1.28)	1.028 (0.81–1.30)	0.831 (0.74–0.93)

*Abbreviation*: *CIEP* counter-immunoelectrophoresis, *IAT* iodine agglutination test

^a^Chi-square values and probabilities (in brackets)

^b^Results of the models after removal of the interaction term. 95% confidence limits are in brackets

**Table 2:** Proportion ± standard deviation of PCR positive blood, spleen, lymph node and bone marrow samples on day 451 post-inoculation for each treatment

Organ	1.5% kelp	0.75% kelp	Control	ϰ^2^_(d.f. = 2)_ (Probability)
Number of mink	19	19	15	
Blood	0.16 ± 0.37	0.16 ± 0.37	0.0 ± 0.0	4.28 (0.12)
Spleen	0.79 ± 0.42	0.58 ± 0.51	0.73 ± 0.46	2.10 (0.35)
Lymph node	0.74 ± 0.45	0.63 ± 0.49	0.60 ± 0.51	0.81 (0.67)
Bone marrow	0.21 ± 0.51	0.26 ± 0.45	0.13 ± 0.35	0.89 (0.64)

### Seroconversion

**Fig. 2:**
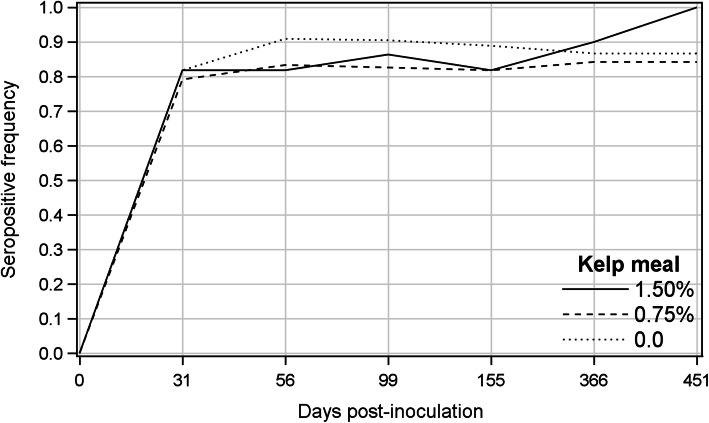
Frequency of seropositive kelp-supplemented and control groups on different post-inoculation days

The incidence of seropositive mink was high for all treatments and showed slight fluctuations over time (Fig. 2). The averages over all treatments were 0.81, 0.85, 0.86, 0.84, 0.87 and 0.91 from 31 to 451 dpi, respectively. Five mink, two from T0.75 and three from T1.5 groups, remained seronegative throughout the course of the study, and two seronegative mink died before the termination of the experiment. All seroconverted animals remained seropositive until 451 dpi. Difference among the treatments and treatment by sampling day interaction were not significant for the proportion of CIEP-positive animals, and the odds ratios for the incidence of CIEP-positive mink were comparable among treatments (Table 1). There was a significant upward trend for the proportion of CIEP-positive animals from 31 until 451 dpi (β = 0.120, odds ratio = 1.127), indicating that for each increment in sampling occasion, there was 12.7% increase in the odds of animals becoming seropositive.

### IAT

**Fig. 3:**
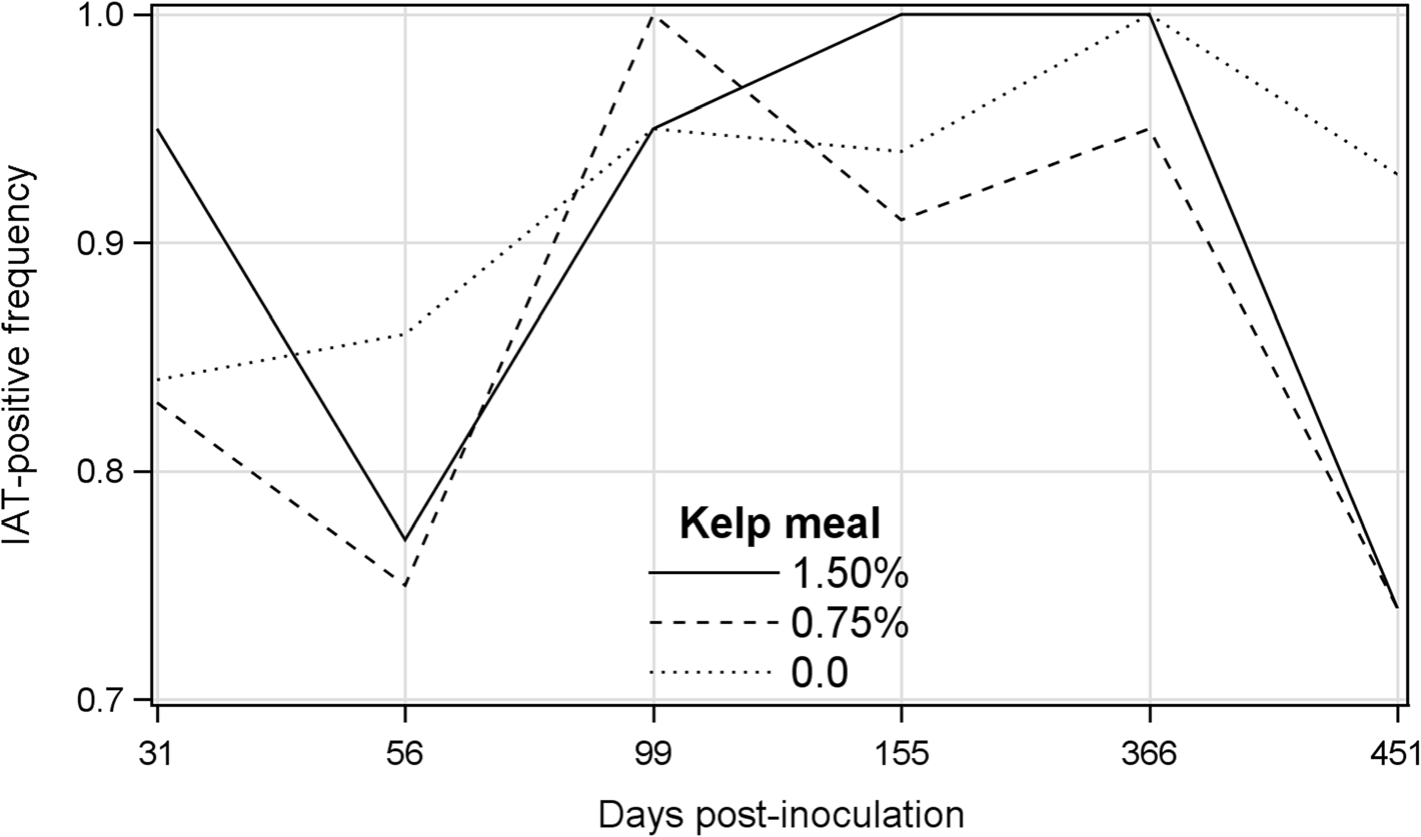
Frequency of positive iodine agglutination test results of the kelp-supplemented and control mink on different post-inoculation days

**Fig. 4:**
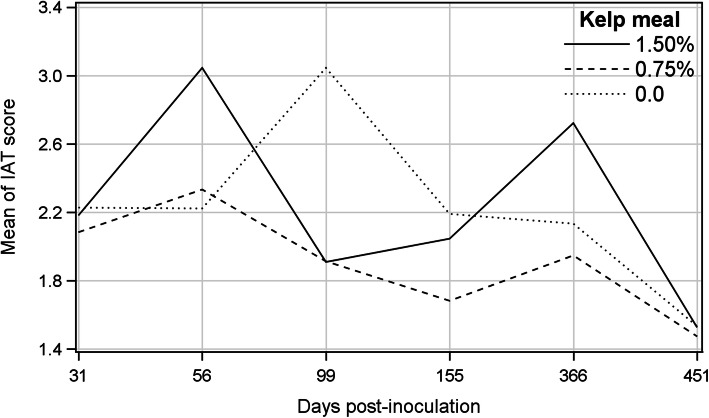
Mean of iodine agglutination test scores (ranged from 0 to 4) of the kelp-supplemented and control mink on different post-inoculation days

Means of percent IAT-positive mink ranged between 73.7% (T0.75 and T1.5 at 541 dpi) and 100% (T0.75 at 99 dpi, T1.5 at 155 and 366 dpi). The frequency of IAT-positive mink in the control group steadily increased from 31 to 451 dpi, whereas it fluctuated on different sampling occasions in kelp supplemented groups and decreased by almost 20% from 366 to 451 dpi (Fig. 3). Differences among treatments, sampling occasions and their interaction were not significant for the percentages of IAT-positive mink (Table 1). Means of IAT scores increased from 31 dpi to their highest levels on 56 dpi for all treatments then gradually declined, and at the termination of the experiment reached the levels lower than those on 31 dpi (Fig. 4). The GENMOD procedure showed no difference among the treatments or treatment by sampling day interaction, but IAT scores significantly declined over time (β = − 0.185, odds ratio = 0.831) (Table 1), indicating that the odds of animals having higher IAT scores decreased by 16.9% for each successive sampling occasion.

### Serum protein measured by a refractometer

**Fig. 5:**
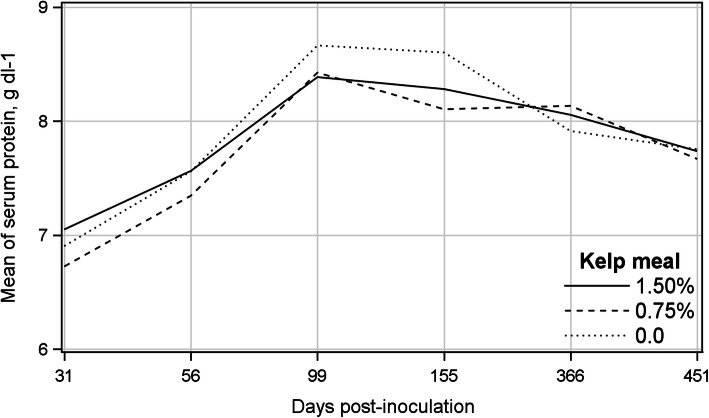
Mean of serum protein measured by a refractometer of the kelp-supplemented and control mink on different post-inoculation days

The means of serum protein of different treatments measured by a refractometer were close to each other at all sampling occasions (Fig. 5) and the Mixed model analysis showed that treatment and treatment by sampling day interaction were not significant, whereas they were significantly changed over time (Table 3). The least-squares mean of serum protein on 31 dpi (6.89 g dl-1) was significantly lower than those on other sampling days, gradually increased by 23.2% to its highest value on 99 dpi (8.49 g dl-1), and then declined until 451 dpi. The least-squares mean on 99 dpi was significantly greater than the values on any other sampling occasion, except on 155 dpi. The level of serum protein on 56 dpi was comparable with that on 451 dpi.

**Table 3:** Least-squares means ± standard errors of total serum protein measured by a refractometer and quantitative ELISA results for treatments and sampling occasions (days post-inoculation)^a^

Measurement	Serum protein, g dl-1	qELISA
% kelp meal	0.40^b^	0.97^b^
1.50	7.91 ± 0.08	0.70 ± 0.05
0.75	7.73 ± 0.08	0.67 ± 0.05
0.0	7.85 ± 0.08	0.71 ± 0.05
Sampling occasion (dpi)	< 0.001^b^	< 0.001^b^
0	–	0.47 ± 0.02 a
31	6.89 ± 0.05 a	-^c^
56	7.49 ± 0.08 b	0.76 ± 0.06 b
99	8.49 ± 0.10 c	0.99 ± 0.06 c
155	8.35 ± 0.12 cd	0.85 ± 0.06 b
366	8.05 ± 0.09 d	0.39 ± 0.04 a
451	7.72 ± 0.09 b	-^c^
Interaction	0.38^b^	0.45^b^

^a^Means followed by different letters are different at *P* < 0.05

^b^Significance level

^c^Samples taken on days 31 and 451 post-inoculation were mixed up at the laboratory

### Antibody titer measured by qELISA

**Fig. 6:**
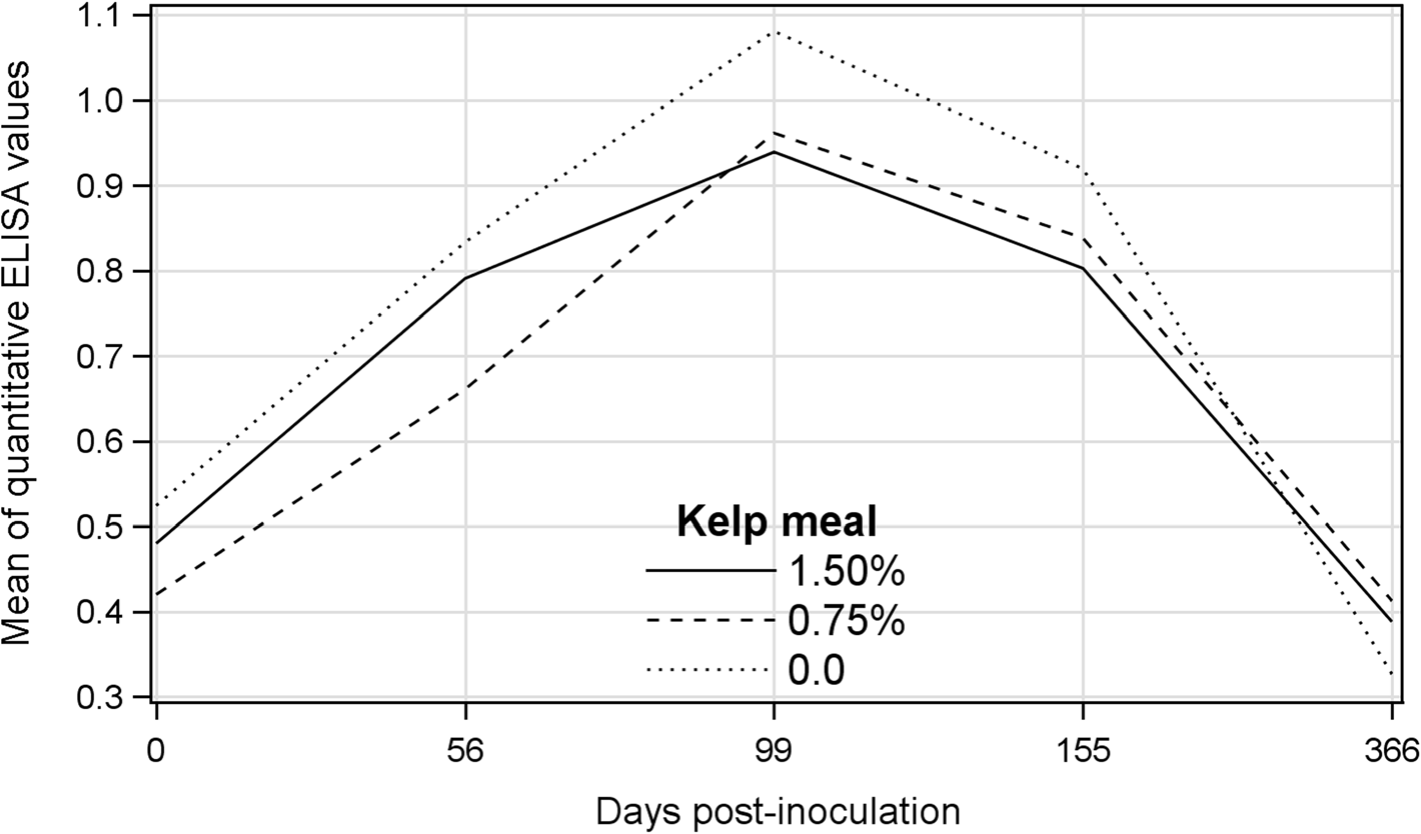
Mean of quantitative ELISA results (unadjusted OD_450_) of kelp-supplemented and control mink on different post-inoculation days

Antibody titers measured by qELISA (unadjusted OD_450_) were generally low and variable among animals within each treatment and among sampling occasions. The means of qELISA readings for all treatments increased from day 0 (pre-inoculation) until 99 dpi, then declined and reached numerically smaller values than the pre-inoculation levels by 366 dpi (Fig. 6). There was no difference among treatments or treatment by sampling day interaction, but changes of qELISA readings over time were significant (Table 3). The mean of qELISA values on 99 dpi was significantly greater than those on other sampling occasions, and that on 366 dpi was the lowest and significantly different from values on the other sampling days. Measurements on 56 and 155 dpi were intermediate and statistically comparable with each other, but significantly different from values on other sampling occasions.

### Associations of total serum protein, IAT score and qELISA over time

The Spearman’s rank correlation coefficients among IAT scores and among serum protein levels measured by a refractometer on different sampling occasions were positive and significant, except those between 31 with 366 and 451 dpi (Table 4), i.e., measurements taken more than 1 year apart. Correlation coefficients between any two successive measures of IAT score (0.54 to 0.71) and between any two successive measures of total serum protein (0.42 to 0.54) were moderate, and the magnitudes declined as time between the measurements increased. There was no relationship between qELISA results prior to inoculation and their subsequent measurements, whereas Spearman’s rank correlation coefficients among measurements after inoculation were all positive and significant, ranging between 0.54 (56 and 366 dpi) and 0.81 (99 and 155 dpi) (Table 5).

**Table 4:** Spearman’s rank correlation coefficients of total serum protein measured by a refractometer (above diagonal) and iodine agglutination test scores (below diagonal) among sampling occasions^a^

DPI	Day 31	Day 56	Day 99	Day 155	Day 366	Day 451
Day 31	–	0.49**	0.38**	0.36**	0.21	0.23
Day 56	0.56**	–	0.53**	0.41**	0.35**	0.44**
Day 99	0.38**	0.67**	–	0.42**	0.60**	0.51**
Day 155	0.30*	0.46**	0.71**	–	0.40**	0.51**
Day 366	0.25	0.57**	0.49**	0.50**	–	0.54**
Day 451	−0.05	0.33*	0.49**	0.54**	0.62**	–

^a^Data pooled over treatments

^**^Significant at *P* < 0.01

^*^Significant at *P* < 0.05

**Table 5:** Spearman’s rank correlation coefficients of quantitative ELISA results on various sampling occasions^a^

Sampling days, pi	Day 56	Day 99	Day 155	Day 366
0	−.06	−.09	−.01	− 010
56	–	0.70**	0.64**	0.54**
99		–	0.81**	0.73**
155			–	0.67**

^a^Data pooled over treatments

^**^Significant at *P* < 0.01

### Associations among serum protein, IAT score and qELISA at the same sampling occasions

The pairwise Spearman’s rank correlation coefficients among the levels of total serum protein measured by a refractometer, IAT scores and qELISA at the same sampling occasion were all positive and significant (Table 6). The estimates were the greatest between total serum protein and IAT scores (0.53 to 0.79), more uniform but small between total serum protein and qELISA (0.36 to 0.48), and correlation coefficients between qELISA and IAT scores steadily declined over time (0.61 to 0.28).

**Table 6:** Spearman’s rank correlation coefficients between total serum protein, IAT score and qELISA on the same sampling occasion

Measurements	Days post-inoculation					
	31	56	99	155	366	451
Serum protein and IAT scores	0.53**	0.74**	0.66**	0.67**	0.59**	0.79**
Serum protein and qELISA	–	0.41**	0.47**	0.36**	0.48**	–
qELISA and IAT score	–	0.61**	0.58**	0.41**	0.28*	–

*Abbreviation*: *IAT* iodine agglutination test, *qELISA* Quantitative ELISA

^**^Significant at *P* < 0.01

### Serum parameters and antibody titer on 451 dpi

Descriptive statistics of antibody titer measured by CIEP and blood parameters at the termination of the experiment for the data pooled over the three treatments are shown in Table 7. Although all measurements significantly deviated from normality, differences between means and medians were rather small, except for ALKP, GGT and the non-transformed antibody titer. Albumin and total protein had the smallest coefficient of variation (8.2 and 8.9%) and GGT had the greatest (232.8%). The results of Kruskal-Wallis tests, median and lower and upper quartiles of the measurement for each treatment are shown in Table 8. Feeding kelp did not have a significant effect on albumin, globulins, albumin/globulin ratio, ALKP, GGT or antibody titer measured by CIEP. Animals supplemented with 1.5% kelp had significantly lower BUN and creatine levels than the controls, and the values for animals supplemented with 0.75% kelp were intermediate and comparable with those of the other groups.

**Table 7:** Descriptive statistics of blood parameters measured by the Chemistry Analyzer and antibody titer measured by CIEP on day 451 post-inoculation (*n* = 53)

Measurement	Mean	Median	Range	CV
Albumin (ALB), g L-1	27.4	28	22–34	8.2
Alkaline phosphatase (ALKP), U L-1	91.8	82	51–336	51.2
Blood Urea nitrogen (BUN), mmol L-1	9.7	8.9	4.9–20.9	34.6
Creatine, μmol L-1	46.2	45	27–90	28.6
Globulins, g L-1	34.9	34	25–52	16.8
Total protein, g L-1	62.4	61	53–80	8.9
Gamma-glutamyl transferase (GGT), g L-1	3.02	0	0–35	232.8
Albumin/Globulin ratio (A/G)	0.81	0.79	0.48–1.24	19.8
Antibody titer^a^	94.0	64	0–256	82.3
Log_2_(titer)^b^	6.5	7	0–9	40.5

*Abbreviation*: *CIEP*, counter-immunoelectrophoresis, *CV* Coefficient of variation

^a^Antibody titer based of the highest plasma dilution which resulted in positive or inconclusive CIEP results

^b^log_2_(titer) + 1 if titer> 0 and 0 if titer = 0

**Table 8:** Median, lower and upper quartiles (in brackets) and the results of Kruskal-Wallis test of differences among treatments for serum parameters measured by the Chemistry Analyzer and antibody titer measured by CIEP on day 451 post-inoculation^a^

Measurement	ϰ^2^_(d.f. = 2)_^b^	1.5% kelp	0.75% kelp	0.0% (control)
Number of mink		19	19	15
ALB	0.42 (*P* = 0.81)	28 (26–29)	28 (26–29)	27 (26–29)
ALKP	0.14 (*P* = 0.93)	82 (64–108)	81 (71–97)	83 (66–98)
BUN	6.40 (*p* = 0.04)	7.5 (6.6–10) a	10.0 (7.5–12) ab	10.8 (8.4–12.9) b
Creatine	6.69 (*p* = 0.03)	36 (34–48) a	46 (38–56) ab	50 (43–52) b
Globulins	0.40 (*p* = 0.82)	33 (29–37)	34 (29–38)	34 (33–39)
Total protein	0.29 (*P* = 0.86)	60 (58–65)	62 (57–65)	61 (59–65)
GGT	4.03 (*P* = 0.13)	0 (0–3)	1 (0–8)	0 (0–1)
A/G	0.46 (*P* = 0.79)	0.84 (0.68–0.96)	0.82 (0.69–0.91)	0.79 (0.73–0.85)
Antibody titer	3.78 (*P* = 0.15)	128 (64–128)	64 (64–128)	64 (32–128)
Log_2_(titer)	3.78 (*P* = 0.15)	8 (7–8)	7 (7–8)	7 (6–8)

^a^Treatment medians followed by different letters are significantly different (*P* < 0.017) when tested by the Mann-Whitney U test

^b^Probabilities are in brackets

The Spearman’s rank correlation coefficients among serum parameters, antibody titer, total serum protein measured by a refractometer and IAT scores recorded at the termination of the experiment are shown in Table 9. Albumin was negatively correlated with globulins (*P* < 0.05) and tended to have a negative correlation with ALKP activity (*P* < 0.10). Positive and significant correlations were observed between ALKP and GGT, BUN and creatine, and between globulins and total serum protein. Creatine also tended (*P* < 0.10) to be positively correlated with globulins and total serum protein. The albumin/globulin ratio was positively and significantly correlated with albumin, but negatively correlated with globulins and total protein. This ratio tended (*P* < 0.10) to be negatively correlated with BUN and creatine. Antibody titer, total serum protein measured by a refractometer and IAT scores were not correlated with any of the serum parameters, except showing positive associations with globulins and total serum protein and negative association with albumin/globulin ratio. Total serum protein measured by a refractometer and IAT scores were positively and significantly correlated with antibody titer.

**Table 9:** Spearman’s rank correlation coefficients among serum parameters, antibody titer, total serum protein and IAT scores on day 451 post-inoculation

Measurement	ALKP	BUN	Creatine	GLOB	TP	GGT	A/G	Titer^a^	Protein^b^	IAT score
ALB	−.25+	−.14	−.04	−.29*	0.11	−.02	0.63**	−.21	−.01	−.20
ALKP	–	0.20	0.09	0.08	−.01	0.33*	−.14	−.09	0.06	0.19
BUN	–	–	0.85**	0.21	0.16	0.08	−.24+	−.08	0.26	0.13
Creatine	–	–	–	0.24+	0.25+	−.01	−.24+	−.03	0.30	0.14
GLOB	–	–	–	–	0.88**	−.02	−.91**	0.57**	0.82**	0.80**
TP	–	–	–	–	–	0.00	−.64**	0.51**	0.85**	0.75**
GGT	–	–	–	–	–	–	0.05	−.16	0.05	0.06
A/G	–	–	–	–	–	–	–	−.53**	−.66**	−.71**
Titer^a^	–	–	–	–	–	–	–	–	0.48**	0.63**

*Abbreviation*: *IAT* iodine agglutination test

^a^Antibody titer based of the highest plasma dilution which resulted in positive or inconclusive CIEP results

^b^Serum protein measured by a refractometer

** Significant at *P* < 0.01

* Significant at *P* < 0.05

^+^ Significant at < 0.10

## Discussion

The observation that there was no difference among treatments for the measurements recorded over time (incidences of positive CIEP, PCR and IAT, the results of qELISA, total protein measured by a refractometer and IAT scores) and the traits recorded at the termination of the study (the proportion of spleen, lymph node and bone marrow samples harboring viral DNA, antibody titer, total serum protein, albumin, globulins and albumin/globulin ratio) suggest that kelp supplementation had no effect on viral replication, viral sequestration or immune response of mink to AMDV infection. To our knowledge, this is the first study on the effect of seaweed supplementation on a non-enveloped DNA virus, and the results contradict the antiviral effects of seaweed supplementation on enveloped viruses in vitro [11–16] and in vivo [7, 17, 18].

The most striking finding of the current study was the significant decrease of creatine and BUN in the mink supplemented with 1.5% kelp compared with the control group, implying that kelp supplementation significantly improved kidney function. The amounts of BUN in mink supplemented with both levels of kelp in the current study were higher than the estimates for healthy brown (16.2 mg dl-1, 5.79 mmol L-1) [22] and healthy dark mink (4.24 mmol L-1) [23], and agrees with a previous report that AMDV-infection significantly elevated its level [24]. Similarly, the amount of BUN in Royal pastel mink which did not show AD symptoms after inoculation with the low pathogenic Pullman strain and sampled from 8 to 126 dpi (12 to 37 mg dl-1, 4.3 to 13.2 mmol L-1) were close to the estimates in the current experiment, but six mink which showed AD symptoms in that study had greater values (23 to 144 mg dl-1, 8.2 to 51.4 mmol L-1) [25]. The current and the above reports clearly suggest that AMDV infection elevates the level of BUN, which is likely the result of trapped immune complexes in the glomeruli [26], which subsequently causes interstitial nephritis and renal dysfunction [1]. The kidneys also showed the greatest severity of AD lesions among organs of naturally infected [27] and experimentally inoculated mink [28]. The enhancement of health, reproduction and survival of the same groups of mink supplemented with 1.5% kelp [29] could have been the result of improved kidney function. The only published report on the effects of AMDV infection on liver function showed that infection did not have a significant effect on ALKP activity, whereas some other measures of liver damage (thymol turbidity, activities of glutamic oxaloacetic transaminase, glutamic pyruvic transaminase) significantly increased [24]. There is not enough information to assess the effects of AMDV infection on liver function with any degree of certainty, and provide a reason for the absence of any effect of kelp supplementation on the markers of liver health in the current study (ALKP and GGT activities).

Reports on the effects of dietary seaweed supplementation on the kidney and liver functions of different animal species are rare. Supplementation of a high-salt diet with a seaweed extract fed to salt-sensitive rats for 7 weeks decreased kidney damage indicated by decreased urinary protein excretion, increased creatinine clearance rate, reduced hypertensive glomerular sclerosis and decreased arterial injury in the kidney [30]. Feeding moderate amounts of a brown seaweed (*Sargassum polycystum)* extract significantly reduced the percentages of necrotic and degenerative liver and kidney cells in experimentally induced diabetic rats, which were attributed to antioxidant pigments or sulphated polysaccharides in the seaweed [31]. Replacing 1.0 and 3.0% of corn with green seaweed (*Ulva lactuca)* meal in a broiler diet in a 21-day study did not have any effect on total serum protein, albumin, globulins, or markers of liver function (AST, CK and GGT), but both seaweed supplements significantly reduced levels of ALT and uric acid compared with the control group [32]. On the contrary, dietary supplementation of ram lambs for 74 days with a green seaweed (*Ulva lactuca*) meal at 3 and 5% of dry matter did not have any effect on the levels of some blood parameters, including total protein, albumin, globulins, AST and ALT, and it was concluded that seaweed had no effect on liver or kidney functions [33]. The same green seaweed meal supplementation at 1 and 2% of dry matter did not have any effect on markers of kidney function (BUN, creatinine, total plasma protein) or liver enzymes (ALKP, AST, ALT) after 56 days of feeding male rabbits and 38 days of feeding pregnant female rabbits [34]. Dietary supplementation of layer hens with 0.5, 1.0% or 2.0% of two species of cultivated red seaweed did not have any effect on the concentration of some blood parameters, including total protein, CK, AST and uric acid during a 30-day feeding trial [35]. The above reports indicate that the effects of seaweed supplementation on kidney and liver functions in unchallenged animals of different species are inconclusive, and the positive effects of kelp on kidney function in the current study was likely because of the role that kidneys play in AMDV pathogenesis in an unknown manner, and deserves further investigation.

The current selection strategy for the establishment of AMDV-tolerant mink herds is based on low antibody titer [36] or low serum gamma globulin [37]. The genetic and biological causes of differences amongst AMDV-infected individuals for antibody titer and serum gamma globulin levels are not clearly understood. If low antibody titer and low gamma globulin level are the result of slow immune response of mink to infection, then long-term selection for low levels of these parameters may weaken the immune system of the mink with some unexpected consequences. If so, it is tempting to hypothesize that improving kidney function, using BUN or creatine levels, could be an alternative approach in selecting tolerant mink.

In agreement with previous reports [28, 38–41], AMDV DNA was detected in the blood by PCR in advance of seroconversion by CIEP, and viremia was short-lived, revealed by the rapid decline in PCR positive cases after 99 dpi. The observation that high percentage of the spleen and lymph node samples harbored the virus, along with a high percentage of seropositive mink at 451 dpi, but much fewer viremic mink at this time, agrees with the previous reports [27, 40–44] and implies that testing blood samples by PCR is not reliable for detecting AMDV infection in chronically infected mink herds. Smaller numbers of PCR positive bone marrow than spleen and lymph node samples agree with a previous report [38] and could have been caused by the method of sampling this tissue.

The observation that antibody production persisted throughout the experiment, even after the termination of viremia, agrees with previous reports for experimentally inoculated mink [25, 28, 38, 40, 41, 45]. Five mink (9.4%) in the current study remained CIEP negative for 451 days, which is a condition observed previously for experimentally inoculated [41] and chronically infected mink [27, 37, 46, 47]. AMDV DNA was detected in the blood and organs of one of these mink (1.9%), indicating that this individual was chronically infected but had low antibody titer throughout its life which was not detectable by CIEP, a finding which agrees with previous reports [27, 37, 42, 48]. The other four seronegative mink were viremic during the early period of the experiment but virus was not detected in their blood at the later dates or in their blood or organs at the termination of the experiment, implying that these animals likely cleared the virus, which was also observed previously [42, 49, 50]. These findings demonstrated that the long-term response of mink to AMDV infection with respect to virus replication and antibody production is complex and combining them in two categories (low and high antibody titers) may decrease the effectiveness of genetic selection for tolerance.

Although IAT has been used for detecting AMDV-infected mink for many years [51], it is not a specific test for AMDV infection because the serum gamma globulin level elevates in response to infection by all pathogens [52]. The incidences of IAT-positive mink in the present study were rather high for all treatments throughout the study (73.7 to 100%), and in agreement with previous studies, fluctuated over time [37, 50, 53]. The fluctuations could be, at least partly, the result of reduced bacterial contamination in feed and the environment during the cold seasons [37, 50]. In the current study, the decline in the incidence of IAT-positive cases from 31 to 56 dpi, which corresponded to October to November, and again from 366 to 451 dpi, corresponded to September to December (Fig. 3), could support this assumption.

Changes in antibody titer measured by qELISA, gamma globulin level measured by IAT score and total serum protein measured by a refractometer were parallel, i.e., increased until 56 or 99 dpi and then declined. The decline in antibody titer and serum gamma globulin level following initial increases were previously observed when animals were monitored for a long time [45, 54]. The declining trends of these parameters could have been the result of reduced viral replication [1] or death of animals with high levels of gamma globulin [25, 49, 55, 56] or high antibody titers [28, 45, 56].

The parallel changes in qELISA, IAT score and serum protein over time were manifested in the positive correlation coefficients among these measurements at different sampling occasions (Table 6). The greatest correlation coefficients were between serum protein measured by a refractometer and IAT scores (0.53 to 0.79) and the lowest were between serum protein and qELISA (0.36 to 0.48), possibly because gamma globulin constitutes a higher proportion of total serum protein than anti-AMDV antibodies. This assumption is supported by the greater correlation coefficients between total serum protein and globulin level (0.82 and 0.88) than between total serum protein and antibody titer (0.48 and 0.51) on 451 dpi (Table 9). The steady decline in the magnitude of correlation coefficients between IAT scores and qELISA over time (0.61 to 0.28) could be caused by the non-linear association between globulins and antibody titer [57], which is the result of declined antibody titer over time.

The positive and rather large correlation coefficients between IAT score and serum globulins (0.80), total serum protein (0.75) and antibody titer (0.63) on 451 dpi (Table 9) point to the efficacy of this low-cost on-farm method for assessing serum globulin level and the state of animal health. The Spearman’s rank correlation coefficients between serum protein levels measured by a refractometer with globulins and serum protein measured by the Chemistry Analyzer (0.82 and 0.85) on 451 dpi were slightly greater than those for IAT scores, but its correlation coefficient with antibody titer was smaller (0.48), suggesting that the refractometer may be less accurate than IAT for assessing animal health. The superiority of IAT over the refractometer was also shown by the higher Spearman’s rank correlation coefficients between successive measures of IAT than serum protein measurements (Table 4).

The greatest Spearman’s rank correlation coefficients between successive measures were for qELISA results compared with IAT and serum protein measured by a refractometer (Tables 4 and 5), suggesting that antibody titer was more stable over time compared with total serum protein or gamma globulin levels. This is contradictory to a previous report that total serum protein was less variable over time compared with antibody titer and gamma globulin level [26]. Means of qELISA readings prior to inoculation (day 0) were numerically larger than those on 366 dpi for all treatments, which could be caused by the lack of adjustment for the background noise in different batches of samples tested by this private laboratory, signifying that this method requires some refinement as previously suggested [58]. Changes in antibody titer over time have previously been reported [26, 45, 54]. In another experiment, antibody titer was stable within 15 days but not when measured in October and the next February [59]. The declining trends in the magnitudes of qELISA, IAT and total serum protein over time, and intermediate correlation coefficients between successive measures of each parameter, suggest that a single measure is not an accurate determinant of the long-term level of these measurements, a point which needs to be taken into consideration when evaluating animals for tolerance to AMDV infection.

The mean and range of albumin in the current study were comparable to the previous estimates for AMDV-infected black mink [57] and slightly lower than the mean of healthy brown female mink (30 g L-1) [22]. Elevated levels of antibody titer and gamma globulin in AMDV-infected mink are associated with a decrease in albumin [24, 60, 61], through reduced albumin synthesis by the liver in order to regulate blood osmotic pressure [62, 63]. The observation that the correlation coefficient between albumin and globulins was negative and significant whereas there was no correlation between albumin and total serum protein, was the manifestation of the homeostatic effect of albumin. It may be concluded that the reduction in the level of albumin is an indication of the severity of hypergammaglobulinemia, regardless of the source of infection, and thus has low diagnostic specificity [63]. The lack of a correlation between albumin and antibody titer in the current and a previous study [57] confirms that albumin level is not an accurate diagnostic tool for AMDV infection.

The level of globulins in the present study was the non-albumin segment of the serum protein, of which gamma globulin is the largest fraction in AMDV-infected mink [60]. The level of globulins at 451 dpi was within the range of the previous estimates for AMDV-infected black mink [57], and also fell within the range of suggested values reported for gamma globulin for AMDV-infected mink, namely between 15 and 50% (30 mg/mL) [1, 61] or between 20 and 63% [55]. Globulin level had a moderate correlation coefficient with antibody titer in the current study (0.57), which was smaller than 0.81 [56] and 0.75 [54] previously reported for the correlation between antibody titer and gamma globulin level. The mean and range of A/G ratio in the current study were close to the previous estimates for AMDV-infected mink [24, 57]. The association between A/G and antibody titer in the current and a previous study [57] was negative and moderate, suggesting that A/G does not have a stronger prognostic advantage than globulin level or antibody titer.

Positive and significant correlation coefficients between the markers of kidney function (BUN and creatine, *r* = 0.85) and between markers of liver function (ALKP and GGT, *r* = 0.33) indicate that the two independent tests for each organ were confirmatory and the results were thus reliable. The levels of BUN, creatine, ALKP and GGT were not associated with any other blood parameters, antibody titer, total serum protein measured by a refractometer or IAT score, implying that the effects of AMDV infection on kidney and liver functions were independent of the immune response of mink to infection. The correlations between ALKP with albumin, BUN with A/G, creatine with total serum protein, globulins and A/G tended to be significant (*P* < 0.10) but difficult to interpret.

## Conclusions

Dietary supplementation with 1.5% kelp meal for 451 days significantly decreased BUN and creatine levels in the blood of mink challenged with AMDV, implying improved kidney function. Kelp supplementation had no effect on markers of liver function, anti-AMDV antibody titer, viral replication or levels of serum protein, suggesting that improved kidney function was independent of viral replication or immune response of mink to infection. Antibody titer measured by qELISA, serum protein measured by a refractometer and gamma globulin level measured by IAT significantly decreased over time, suggesting that measurement at a single point in time may not be an accurate predictor of these measurements. Further research is needed to validate the effects of kelp supplementation on improved kidney function.

## Methods

### Source of seaweed

*Ascophylum nodosum* (kelp) was hand harvested and prepared by Tidal Organics (http://tidalorganics.com/) as previously described [29].

### Source of animals, animal management and experimental design

Animal management and experimental design were previously described [29]. In brief, 75 five-month old female black American mink (*Neovison vison*) were obtained in September 2013 from an AMDV-free private farm in Nova Scotia, transferred to the Aleutian Disease Research Center and were kept in individual cages. Three days after arrival, animals were anesthetized and intranasally inoculated with 60 μL of a 10% (W/V) passage 2 of a local strain of AMDV as previously reported [41]. The diet gradually changed from the wet feed used on the farm of origin to a commercial dry pellet (National Feeds Inc., Maria Stern, OH, USA). Animals were randomly divided into three groups and the kelp meal added at the rates of 1.5% (T1.5), 0.75% (T0.75) or 0% (T0, control) of the feed. The pellet’s nutritional composition changed based on the production cycles of the mink. Feeds were added to feeders as needed, and animals had free access to the feed and water. Some kelp particles settled out in feeders, which were not recorded.

### Sampling

On days 0 (before inoculation, Sep. 9, 2013), 31 (Oct. 10), 56 (Nov. 5), 99 (Dec. 17), 155 (Feb. 11, 2014), 366 (Sep. 9) and 451 (Dec. 4) post-inoculation animals were sedated and blood was collected by toenail cutting into heparinized capillary tubes for plasma preparation for the CIEP test, in EDTA-coated capillary tubes for viral detection by PCR, in plain tubes for serum preparation for IAT and for measuring total serum protein, and on absorbent combs for measuring antibody titer by qELISA. Animals which survived until December 4, 2014 (451 dpi) were euthanized and blood was collected by heart puncture as explained above. Samples of spleen, mesenteric lymph nodes and bone marrow were harvested and transferred into cryovials for detection of AMDV DNA by PCR. Bone marrow was flushed out of the tibia with 0.5 ml of PBS. Blood samples were kept in a refrigerator overnight and centrifuged at 1397 g (Porta Spin C826 centrifuge, Unico, Dayton, NJ, USA) for 10 min for plasma and serum preparation. Serum, plasma and tissues were stored at -80 °C until use. Sample preparation was performed at the bio-secure laboratory (Level II) following the approved Standard Operating Procedures.

### Laboratory procedures

The CIEP test [46] was performed by the Animal Health Laboratory of the Nova Scotia Department of Agriculture in Truro, Nova Scotia, Canada, which is an accredited laboratory for this test under the Standards Council of Canada. The antigen was from the Research Foundation of the Danish Fur Breeders Association, Glostrup, Denmark. In cases where bands on gels were faint, the results were reported as doubtful by the laboratory. The doubtful results were likely caused by low antibody titers, and were considered as positive. Direct AMDV DNA amplification was performed by PCR on plasma and cell-free tissue homogenates in 25 μL total volumes containing (final concentration) 1X PCR buffer, 0.2 mM each dNTP, 400 nM each primer, 1X (12.5 μL) PEC-2 Enhancer and 0.25 μL of Omni Klentaq-LA (DNA Polymerase Technology, http://www.klentaq.com) as previously described [64] with the primer pair 60F/60R [65]. Three PCR tests were performed on each sample using 0.5, 1.0 and 2.0 μL of plasma or tissue homogenate. This battery of tests was repeated when there was one faint band or no amplification. A sample was declared PCR positive when at least one clearly visible band or at least two faint bands were observed on the gel. The sample was considered negative when none or one of the six reactions produced a faint amplification. All PCR tests included plasma from an AMDV-infected mink (positive control) and a blank reaction (negative control). To avoid cross-contamination, sample preparation, DNA extraction, PCR amplification and PCR product testing were performed in four different laboratories with unidirectional sample movement. Sterile filtered-tips were used throughout the experiment.

qELISA was performed at the Middleton Veterinary Services, Middleton, NS (http://www.greenwoodanimalhospital.ca). Blood-soaked combs were air dried for 1 h and kept in a refrigerator before sending to the laboratory at ambient temperatures and tested for antibody titer as previously reported [58]. Total serum protein was measured by a refractometer (Reichert TS 400, Reichert Analytical Instruments, Depew, NY, USA). Measurements were taken at room temperature by a single technician, and the instrument was tarred with distilled water before each measurement. For the IAT test, between 10 and 30 μl (one drop) of serum were put on a glass slide, to which an equal volume of the home-made Lugol’s Solution (2 g iodine and 4 g potassium iodide in 28.35 mL distilled water) was added, mixed with a toothpick, and the results were recorded within 3 min. Scores were 0 (clear brown solution), 1 (a few small-size precipitates which appeared after a few minutes of swirling by hand), 2 (many small-size precipitate), 3 (heavy precipitate after mixing), and 4 (immediate formation of heavy dark brown precipitate) [51].

In addition to the fresh plasma samples that were tested by CIEP, frozen plasma samples collected at the termination of the experiment were thawed at room temperature, two-fold serially diluted 10 times (1/2 to 1/1024) with PBS and tested by CIEP. The titer of anti-AMDV antibody was recorded as the reciprocal of the highest dilution of plasma that showed positive or doubtful results. Total serum protein, albumin, BUN, creatine, globulins, ALKP and GGT were measured using the Vet-Test Chemistry Analyzer (IDEXX International, http://www.idexx.dk/smallanimal/inhouse/vetlab/vettest-chemistry.html).

### DATA analysis

Data were analyzed with SAS V9.4 for Windows (SAS Institute Inc., Cary, NC). Prior to analyses, data were checked for normality by the Shapiro-Wilk test implemented in the UNIVARIATE procedure. Linear logistic regression models using a Generalized Estimating Equation (GEE) algorithm in the GENMOD procedure with the independent correlation structure, a binomial distribution and the logit link function were used to assess differences among treatments for the incidence of positive CIEP, PCR and IAT results. The fixed effects of treatment, sampling occasions (continuous variable 1, 2, 3, etc.) and their interactions were included in the models. The interactions and quadratic terms of sampling occasions were not significant and were removed from the final models. The random effect of individual mink was used in the REPEATED statement to take care of the correlation among measurements recorded over time on the same mink. Pairwise comparisons of treatments were made using the ESTIMATE statement in GENMOD. The odds ratios and their 95% confidence limits are reported. The ordinal measures of IAT scores (0 to 4) were analyzed using the same model as above but with a multinomial distribution and the cumulative logit link function.

Linear mixed models (PROC MIXED) were used to test the differences among treatments, sampling days and their interactions for qELISA results and serum protein measured by a refractometer. The REPEATED statement was used to take into account the repeated observations on animals over time. The appropriate correlation structures were determined after fitting five models with different correlation structures and selecting the model with the smallest BIC value. The smallest BIC values were obtained by the unstructured correlation for qELISA results and heterogeneous autoregressive for serum protein, which were used in the final analyses. Restricted maximum likelihood estimation and Type 3 tests of fixed effects were used with the Kenward-Roger method. Post hoc comparisons among least-squares means were performed with the Tukey-Kramer adjustment.

Measurements taken at the termination of the experiment on 451 dpi, including serum parameters (albumin, globulins, albumin:globulin ratio, total protein, BUN, creatine, ALKP, GGT), and antibody titer measured by CIEP deviated from normality and treatment effects were tested by the non-parametric Kruskal-Wallis test. Antibody titers were transformed to log_2_(CIEP) + 1 if CIEP> 0 and 0 if CIEP = 0 prior to analysis. In cases where this test was significant at α < 0.05, pairwise comparison of treatment means was performed by the Mann-Whitney U test with Bonferroni correction (α = 0.05/3 = .017). Results are presented as median and upper and lower quartiles. The Likelihood Ratio Chi-square test was used to compare treatments for the proportion of animals which were PCR positive in the blood and organs at the termination of the experiment. The associations among the measurements were calculated by the Spearman’s rank correlation.

## Data Availability

Data will be provided by the corresponding author upon request.

## Abbreviations

AD: Aleutian disease
ALKP: Alkaline phosphatase
ALT: Alanine aminotransferase
AMDV: Aleutian mink disease virus
AST: Aspartate aminotransferase
BUN: Blood urea nitrogen
CIEP: Counter-immunoelectrophoresis
CK: Creatine kinase
dpi: day post-inoculation
GGT: Gamma-glutamyl transferase
IAT: Iodine agglutination test
PBS: Phosphate buffered saline
PCR: Polymerase chain reaction
qELISA: Quantitative enzyme-linked immunosorbent assay
